# SOD-1/2 Involvement in the Antioxidant Molecular Events Occurring upon Complex Magnetic Fields Application in an *In Vitro* H_2_O_2_ Oxidative Stress-Induced Endothelial Cell Model

**DOI:** 10.3390/ijms26178600

**Published:** 2025-09-04

**Authors:** Alessia Ricci, Susi Zara, Viviana di Giacomo, Marialucia Gallorini, Monica Rapino, Natalia Di Pietro, Alessandro Cipollina, Adriano Piattelli, Amelia Cataldi

**Affiliations:** 1 Department of Pharmacy, “G. d’Annunzio” University of Chieti-Pescara, 66100 Chieti, Italy; alessia.ricci@unich.it (A.R.); susi.zara@unich.it (S.Z.); viviana.digiacomo@unich.it (V.d.G.); marialucia.gallorini@unich.it (M.G.); 2 Unit of Chieti, Genetic Molecular Institute of CNR, “G. d’Annunzio” University of Chieti-Pescara, 66100 Chieti, Italy; monica.rapino@unich.it; 3 Department of Medical, Oral and Biotechnological Sciences, “G. d’Annunzio” University of Chieti-Pescara, 66100 Chieti, Italy; natalia.dipietro@unich.it; 4 Independent Researcher, 92019 Sciacca, Italy; alexandros1960@libero.it; 5 School of Dentistry, UniCamillus-Saint Camillus International University of Health and Medical Sciences, 00131 Rome, Italy; apiattelli51@gmail.com; 6 Ud’A Techlab, “G. d’Annunzio” University of Chieti-Pescara, 66100 Chieti, Italy

**Keywords:** endothelial cells, Complex Magnetic Fields, ROS, antioxidants, cell turnover, SOD-1/2, Nrf2

## Abstract

Endothelial function plays a key role in tissue repair. Reactive Oxygen Species (ROS) production impairs tissue renewal and homeostasis. Complex Magnetic Fields (CMFs) have been attracting attention as a non-invasive tool to promote tissue regeneration, especially through angiogenic stimulation. The present study aims to investigate CMF effect in an *in vitro* model of oxidative stress-stimulated Endothelial Cells (ECs). Cells were pre-treated with H_2_O_2_ to mimic an oxidative environment, followed by the application of three CMF programs repeated in two experimental sets: two consecutive cycles (two cycles) or two cycles spaced 24 h apart (T0+T24). Flow cytometry investigation shows that both CMF applications reduce ROS production, presumably promoting SODs proteins expression. Specifically, two cycles affect mitochondrial SOD-2 expression, which may promote cellular turnover by upregulating pro-apoptotic proteins, leading to mild cell death balanced with increased cell viability. T0+T24 application promotes cytosolic SOD-1 expression, which may influence the expression and release of antioxidant molecules, as evidenced by the increased protein levels of Akt/Nrf2 and the overall antioxidant activity measured post-treatment. In conclusion, ROS-induced EC dysfunction can be reverted by CMF application: 2 cycles could be applied when cellular renewal is required (such as in pathological wounds) while T0+T24 could be useful when an antioxidant and anti-inflammatory effect is needed (e.g., in edema or muscular lesions).

## 1. Introduction

Blood vessels, being a critical interface between the circulation and the different organs, exert their function on tissue homeostasis and adaptation to pathological conditions. Indeed, endothelium injury plays a critical role in more than 80 diseases, including atherosclerosis, stroke, peripheral limb ischemia, cancer, vascular malformations, and many other conditions [1]. At the same time, the vascular system exerts an essential activity in the healing promotion of the surrounding damaged tissues [2]. One of the main causes of endothelial impairment is represented by dysregulated Reactive Oxygen Species (ROS) production, defined as oxidative stress: by influencing signaling pathways that control cell fate and function, ROS promote aberrant redox signaling and cell injury, causing inflammation and endothelial dysfunction that lead to vascular damage and cardiovascular remodeling [3,4]. In the occurrence of endothelial injury, a predominant role is covered by mitochondria, the primary site of cellular aerobic respiration and organelles responsible for energy information transfer and ROS generation. Indeed, mitochondrial membrane dysfunction often determines the evolution of endothelial cell injury towards necrosis or apoptosis [5]. Antioxidant molecules are essential in counteracting the increasing levels of ROS, keeping the system in a balanced state. Among them, Superoxide Dismutases (SODs) are antioxidant metalloenzymes mainly involved in the cellular defense against oxidative stress. SODs catalyze the conversion of superoxide anion free radical in hydrogen peroxide and molecular oxygen. The two main isoforms of SODs are SOD-1 and SOD-2: SOD-1 is predominantly a cytosolic isoform, while SOD-2 is a mitochondrial isoform [6]. Based on stressful conditions and oxidative stress intensity, SODs production and activity can be linked to other cellular responses. Indeed, one SOD-mediated cellular mechanism to maintain homeostasis is the promotion of cellular turnover, which often occurs through apoptotic processes [7]. Excessive ROS levels, along with a reduction in mitochondrial membrane potential (MMP) and increased mitochondrial permeability, determine apoptotic pathways recruiting, triggering a caspase’s cascade activation, which culminates in the apoptotic event [8,9]. Although apoptosis is generally a process aimed at maintaining cellular turnover, in specific conditions it can be altered due to abnormalities in the regulation of cell death as a major contributing factor to the development of diseases [10]. Mitochondrial SOD-2 activation in response to oxidative stress can control the apoptotic event, promoting cellular turnover [11]. A second possible SOD-mediated mechanism to maintain a balanced homeostasis within the cells when oxidative stress occurs is through antioxidant pathways that can converge on the apoptotic event, blocking or delaying the process, thus promoting cellular renewal across other pathways. For instance, in endothelial cells (ECs) exposed to *Eucommia ulmoides* olive leaves, apoptosis is attenuated by upregulating Akt/Nrf2 signaling and downregulating oxidative stress [12]. Indeed, an additional regulator of the antioxidant responses is represented by Nuclear erythroid 2–related factor 2 (Nrf2) transcription factor responsible for the control of the antioxidant defense system [13], thereby safeguarding ECs against ROS [14,15]. Nrf2/SOD signaling pathway could be Akt-mediated, managing oxidative stress and apoptosis in several cell models [16].

The use of Complex Magnetic Fields (CMFs) is a non-invasive therapeutic approach characterized by low and very low frequency and intensity magnetic fields that offer a promising medical alternative thanks to their potential anti-inflammatory and antioxidant effects. Studies have shown that CMFs can decrease the production of ROS and reduce the expression of key pro-inflammatory mediators [17,18]. CMFs are emitted by an electronic device and are composed of pulse multifrequency electromagnetic fields between 1 and 250 µT, variable in intensity, frequency, complex waveform, and time stimulation [19]. Moreover, CMFs disclose therapeutic potential in reparative and regenerative processes, with a positive effect on ECs promoting different steps of angiogenesis [20], as well as in neurodegenerative diseases treatment [21]. Thus, considering the crucial role played by oxidative stress in the health of the vascular system and that CMFs represent a valid, alternative and non-invasive tool to promote angiogenesis, here we aimed at investigating the antioxidant molecular mechanisms recruited after CMF exposure in an *in vitro* model of oxidative stress-stimulated ECs.

## 2. Results

### 2.1. Effect of CMFs Application on H_2_O_2_-Mediated ROS Production, Metabolic Activity and Cytotoxic Response in EA.hy926 Cells

**Figure 1:**
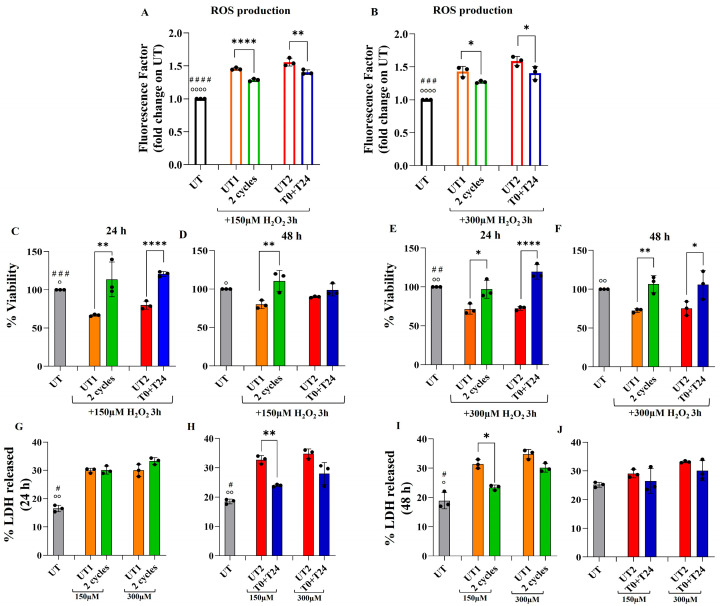
Intracellular ROS generation (**A**,**B**), MTT test (**C**–**F**), LDH (%) released (**G**–**J**) in EA.hy926 endothelial cells after 3 h of 150 µM or 300 µM H_2_O_2_ stimulation followed by CMF application. (**A**,**B**): ROS generation was measured by flow cytometry 24 h after 2 cycles and T0+T24 stimulation. Histograms represent median values ± SD of three independent experiments in which the mean fluorescence intensity (MFI) generated by the oxidation of H_2_DCFDA is reported as fold change on UT. One-way ANOVA followed by Tukey’s test: °°°° *p* < 0.0001 between UT and UT1; #### *p* < 0.0001 between UT and UT2; **** *p* < 0.0001; ** *p* < 0.01; * *p* < 0.05. (**C**–**F**): MTT analysis was performed 24 h (**C**,**E**) and 48 h (**D**,**F**) after CMFs stimulation. Metabolic activity of UT1 and UT2 is normalized on UT; metabolic activity of 2 cycles treatment is normalized on UT1; metabolic activity of T0+T24 is normalized on UT2. Data shown represent the mean ± SD of three independent experiments One-way ANOVA followed by Tukey’s test: °° *p* < 0.01, ° *p* < 0.05 between UT and UT1; ### *p* < 0.001, ## *p* < 0.01 between UT and UT2; **** *p* < 0.0001; ** *p* < 0.01; * *p* < 0.05. (**G**–**J**): LDH release percentage was estimated 24 h (**G**,**H**) and 48 h (**I**,**J**) after CMFs stimulation. Data are normalized on MTT results and are represented as the mean ± SD of three independent experiments. One-way ANOVA followed by Tukey’s test: °° *p* < 0.01, ° *p* < 0.05 between UT and UT1/UT2 (150µM H_2_O_2_); # *p* < 0.05 between UT and UT1/UT2 (300 µM H_2_O_2_); ** *p* < 0.01; * *p* < 0.05.

EA.hy926 cells were treated with two different concentrations of H_2_O_2_ (150 µM and 300 µM) for 3 h in order to establish an oxidative stress condition. Subsequently, cells were exposed to three CMF programs: A. anti-stress; B. anti-inflammatory; C. regenerative tissue. The effect of CMFs combined treatment (A+B+C) on H_2_O_2_-stimulated ECs was investigated in terms of total ROS production through a flow cytometry analysis of H_2_DCFDA positive cells. First of all, 3 h of stimulation with both H_2_O_2_ concentrations (150 and 300 µM) increases ROS levels compared to ECs that are not H_2_O_2_-stimulated (UT): indeed, a statistically significant increase in ROS production is recorded comparing UT with both UT1 and UT2, in the presence of 150 µM (Figure 1A) or 300 µM of H_2_O_2_ (Figure 1B), suggesting that both conditions are adequate to induce an oxidative stress environment. The application of CMFs reduces the amount of ROS produced in both H_2_O_2_-stimulated models: when H_2_O_2_ 150 µM is applied for 3 h (Figure 1A), 24 h after two cycles, a significant reduction in ROS production is detected compared to UT1, and a similar trend is also recorded 24 h after T0+T24 application, with a statistically significant reduction in total ROS with respect to UT2. When H_2_O_2_ 300 µM is applied for 3 h (Figure 1B), the reduction in ROS levels after CMFs application is still evident: in fact, a reduction of H_2_DCFDA positive cells is detected after both the two cycles and T0+T24 stimulation were compared to UT1 and UT2, respectively.

To understand the effect of ROS production on cell metabolic activity as indirect information of cell viability, MTT analysis was performed. MTT test supports the data obtained from ROS measurement: both concentrations of H_2_O_2_, 150 µM (Figure 1C,D) and 300 µM (Figure 1E,F), administered for 3 h, are adequate to induce an effect on ECs, with around 30% reduction in UT1 and UT2 ECs metabolic activity compared to UT (not H_2_O_2_-stimulated condition), creating a stressful environment for the cells, but without a strong impairment of cell viability. In the first condition (3 h of 150 µM H_2_O_2_, Figure 1C), 24 h after CMF stimulation, it is evident a statistically significant increase in EC metabolic activity comparing UT1 with two consecutive cycles and even stronger comparing UT2 with T0+T24. After 48 h, the significant increase in EC metabolic activity in the two cycles’ conditions is still persisting when compared to UT1, while UT2 reaches approximately T0+T24 levels with no statistically significant changes between them (Figure 1D). In the second oxidative stress condition (3 h of 300 µM H_2_O_2_, Figure 1E), a similar trend in cell metabolic activity variation is disclosed, with a significant increase 24 h after the application of the two cycles compared to UT1 and T0+T24 application compared to UT2; the increment also persists in both experimental stimulations after 48 h (Figure 1F).

LDH release was further evaluated in order to support MTT results. Again, the stressful conditions, due to 150 µM and 300 µM H_2_O_2_ administration, also affect cell cytotoxicity, with an increased amount of LDH released in UT1 and UT2 compared to UT. Considering the first experimental set (two cycles, Figure 1G,I), 24 h after CMF stimulation, the amount of LDH released after both H_2_O_2_ pre-treatments is comparable in UT1 and the two cycles, with no statistically significant changes (Figure 1G). After 48 h (Figure 1I), a significant reduction in LDH released is detected comparing UT1 and the two cycles pre-treated with 150 µM H_2_O_2_, but no significant changes are recorded after 300 µM H_2_O_2_ pre-treatment. Considering the second experimental set, T0+T24 stimulation (Figure 1H,J), 24 h after CMFs, in the pre-treated condition with 150 µM H_2_O_2,_ a statistically significant LDH release reduction is recorded comparing UT2 with T0+T24, maintaining the same trend also in 300 µM H_2_O_2_ pre-treated condition; however, without a statistically significant change (Figure 1H). On the contrary, 48 h after T0+T24 stimulation, no significant change is recorded in LDH release upon both H_2_O_2_ concentrations (Figure 1J).

All the above reported results suggest that the first oxidative pre-treatment (150 µM H_2_O_2_ for 3 h) is adequate to activate stressful stimulus within the cells, as also reported in the literature [22,23], in order to investigate the molecular and biological events occurring upon CMF treatment.

### 2.2. Effect of CMFs on SOD1/2 Protein Expression

**Figure 2:**
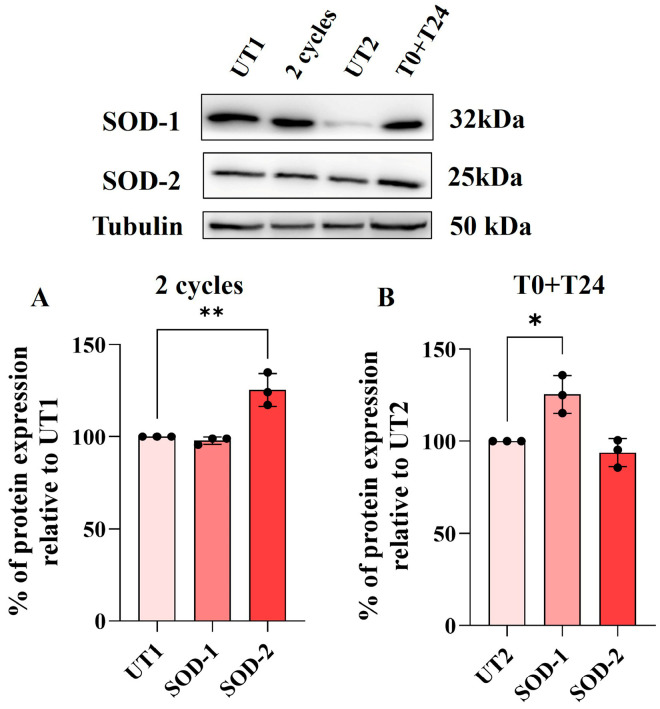
Western blotting analysis of SOD-1 and SOD-2 expression levels in EA.hy926 cell line after H_2_O_2_ pre-treatment followed by CMFs stimulation. Cells were pre-treated for 3 h with 150 µM H_2_O_2_ followed by 2 cycles (**A**) or T0+T24 stimulation (**B**). Histograms display densitometric protein levels of SOD-1 and SOD-2 24 h after CMFs stimulation obtained from the immunoreactive bands analysis and are expressed as mean ± SD of three independent experiments, normalized on loading control (tubulin) and reported as relative to the specific UT (2 cycles on UT1—T0+T24 on UT2). Unpaired *t* test vs. UT1 or UT2: ** *p* < 0.01; * *p* < 0.05.

Cytosolic SOD-1 and mitochondrial SOD-2 protein expression were then assessed in order to understand how CMF application could counteract oxidative stress obtained through 3 h of 150 µM H_2_O_2_ pre-treatment. A statistically significant increase in mitochondrial SOD-2 expression is disclosed 24 h after two cycle stimulations, compared to UT1, while no changes are evident in SOD-1 expression (Figure 2A). On the contrary, 24 h after T0+T24 stimulation, a substantial increase in SOD-1 protein expression is detected compared to UT2, with no statistically significant changes in SOD-2 (Figure 2B).

### 2.3. Effect of CMFs on EC Turnover Stimulation upon H_2_O_2_-Induced Oxidative Stress

**Figure 3:**
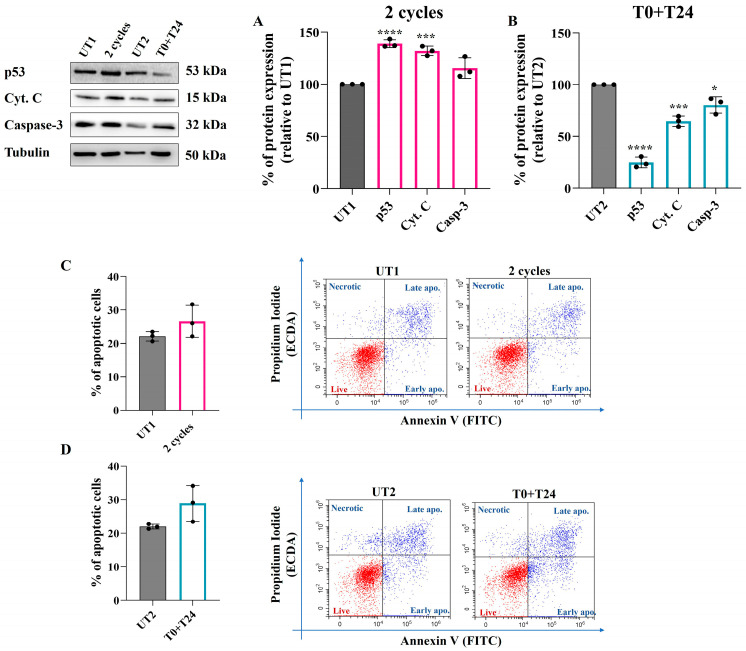
Western blotting analysis of pro-apoptotic proteins (**A**,**B**) and flow cytometry analysis of apoptosis induction (**C**,**D**) in EA.hy926 cell line after 3 h of 150 µM H_2_O_2_ pre-treatment followed by 2 cycles or T0+T24 CMF stimulation. (**A**,**B**): Histograms display densitometric values of each protein 24 h after CMFs stimulation obtained from the immunoreactive bands analysis and expressed as mean ± SD of three independent experiments, normalized on loading control (tubulin) and reported as relative to the specific UT (2 cycles on UT1—T0+T24 on UT2). Unpaired *t* test vs. UT1 or UT2: **** *p* < 0.0001; *** *p* < 0.001; * *p* < 0.05. (**C**,**D**): Percentages of Annexin/PI-stained cells analyzed by flow cytometry are shown 48 h after 2 cycles (**C**) and T0+T24 (**D**) stimulation. Histograms represent the percentage of total apoptotic cells (Annexin V+/PI+) reported as mean ± SD of three independent experiments. Representative dual-parameter fluorescence density dot plots are displayed close to the histograms. Unpaired *t* test.

To better elucidate the molecular pathways activated by CMFs upon a 150 µM H_2_O_2_ pre-treatment and considering that ROS production within the cells could exert a pro-apoptotic effect, Western blotting analysis of some pro-apoptotic markers was performed. A statistically significant increase in p53 and Cytochrome C proteins expression 24 h after two cycle stimulations is recorded compared to UT1, while Caspase-3 protein level increases with respect to UT1, even though this increment is not statistically significant (Figure 3A). On the contrary, 24 h after T0+T24 CMFs application, a significant reduction in p53, Cytochrome C, and Caspase-3 protein levels compared to UT2 is detected (Figure 3B).

Then, to assess the role of these molecules on apoptosis induction, a flow cytometry analysis of Annexin/PI positive cells was performed. A slight increase in Annexin+/PI+ cells amount is detected 48 h after both the two cycles (Figure 3C) and T0+T24 (Figure 3D) with respect to UT1 and UT2, respectively, even though, in both conditions, data are not statistically significant.

**Figure 4:**
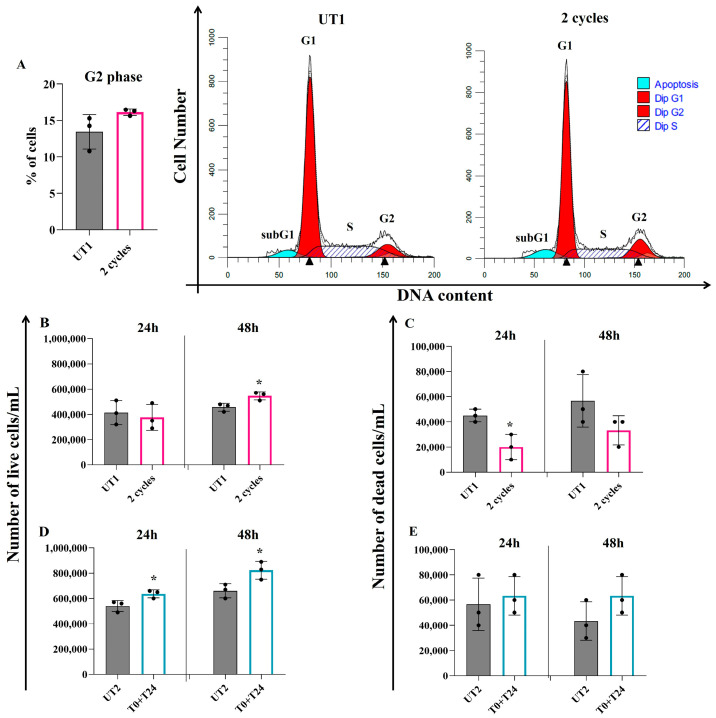
Cell cycle profile (**A**) and Trypan blue exclusion assay (**B**–**E**) of EA.hy926 endothelial cell line after 3 h of 150 µM H_2_O_2_ pre-treatment followed by 2 cycles or T0+T24 CMF stimulation. (**A**): on the left histogram represents the percentage of cells in G2 phase 24 h after 2 cycle stimulations, analyzed by flow cytometry after PI staining; on the right cell cycle profiles represented by fluorescence emission peaks obtained after PI staining (*y*-axis = cell count; *x*-axis = PI fluorescence emission in the FL-channel directly proportional with DNA content). (**B**–**E**): number of live (**B**,**D**) and dead cells (**C**,**E**) 24 and 48 h after 2 cycles (**B**,**C**) and T0+T24 (**D**,**E**) stimulations. Histograms show mean ± SD of three independent experiments. Unpaired *t* test: * *p* < 0.05.

In addition, 24 h after two consecutive cycles, cell cycle modulation is recorded, with an increased percentage of cells in G2 phase compared to UT1, even if data are not statistically significant (Figure 4A). Regarding T0+T24 stimulation, no changes are evident in cell cycle analysis.

To corroborate data previously obtained with the Annexin/PI analysis, Trypan Blue exclusion assay was performed 24 and 48 h after CMFs stimulation. The number of live cells 24 h after two CMF cycles of pre-treated ECs is not influenced in a statistically significant manner compared to UT1; it is necessary to reach 48 h after CMF stimulation to record a significant increase in the number of live cells compared to UT1 (Figure 4B). On the contrary, looking at the same experimental condition, 24 h after 2 cycles the number of dead cells substantially and significantly decreases with respect to UT1, with no significant changes after 48 h (Figure 4C). T0+T24 condition influences the number of live cells starting from 24 h after the stimulation, with a significant increase with respect to UT2; this increase persists also after 48 h (Figure 4D). The number of dead cells shows an increased trend compared to UT2 after both time points of stimulation (24 and 48 h); however, these data are not statistically significant (Figure 4E).

### 2.4. Effect of CMFs on H_2_O_2_-Stimulated EC Migration

**Figure 5:**
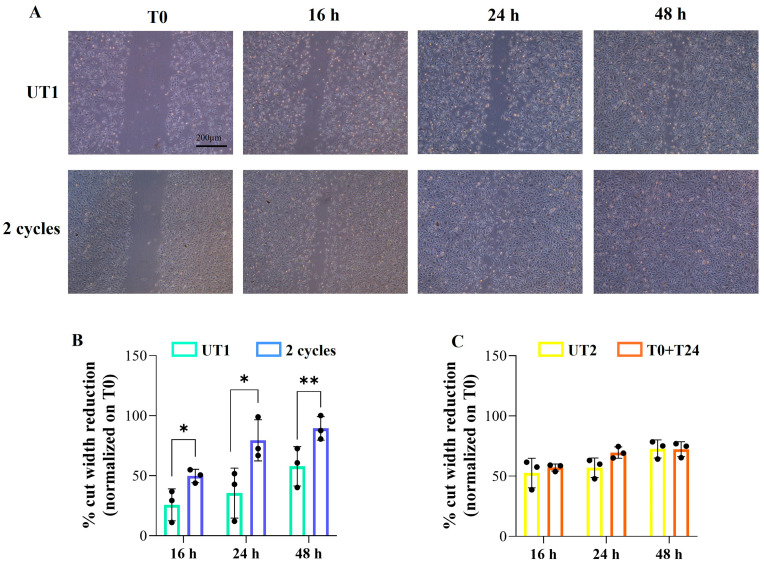
Migration assessment of EA.hy926 cell line after H_2_O_2_ pre-treatment followed by CMF stimulation. Cells were pre-treated for 3 h with 150 µM H_2_O_2_ followed by 2 cycles or T0+T24 stimulation. (**A**): Representative images of scratch wound-healing assay performed at T0 (starting point, when cell monolayer was wounded) and at 16, 24, and 48 h after 2 cycle stimulations. (**B**,**C**): Histograms displaying the cut width reduction % obtained from scratch wound-healing assay at the specified experimental time points after 2 cycles (**B**) and T0+T24 (**C**) stimulation. Quantification was performed by using Fiji 2.1.0/1.53o ImageJ software. Cut reduction 14, 24 and 48 h after stimulation is normalized on T0 and data are presented as mean ± SD of three independent experiments. Unpaired *t* test: ** *p* < 0.01; * *p* < 0.05.

In order to further support the idea that CMF application is suitable for tissue regeneration, a wound-healing assay was performed considering the importance of cell migration during this event. The starting point to evaluate the migratory capability of cells is cell monolayers scraped before CMF application (T0). At all three time points evaluated, 16 h, 24 h, and 48 h after two cycle stimulations, ECs migration constantly and significantly increases compared to UT1, with a complete wound closure 48 h after CMFs stimulation (Figure 5A,B), whereas when T0+T24 is applied, no statistically significant changes are recorded in terms of cell migration compared to UT2 (Figure 5C).

### 2.5. Effect of CMFs on the Antioxidant Factors Expression in H_2_O_2_-Stimulated ECs

**Figure 6:**
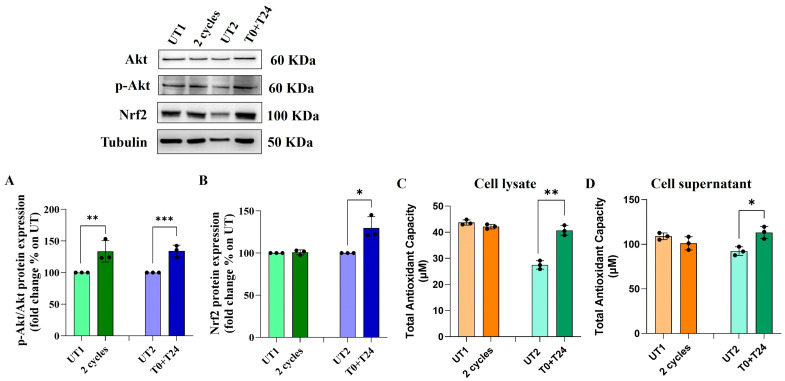
Western blotting analysis of p-Akt/Akt ratio (**A**) and Nrf2 (**B**) expression levels and TAC analysis (**C**,**D**) in EA.hy926 cell line after H_2_O_2_ pre-treatment followed by CMF stimulation. Cells were pre-treated for 3 h with H_2_O_2_ 150 µM followed by 2 cycles or T0+T24 stimulation. (**A**,**B**): Histograms displays densitometric values of p-Akt/Akt ratio (**A**) and Nrf2 (**B**) 24 h after CMFs stimulation obtained from the immunoreactive bands analysis and are expressed as mean ± SD of three independent experiments, normalized on loading control (tubulin) and reported as relative to the specific UT (2 cycles on UT1—T0+T24 on UT2). (**C**,**D**): Histograms displays TAC measured in cell lysate (**C**) and in cell supernatants (**D**) 48 h after CMFs stimulation. Values were normalized on MTT results and reported as mean ± SD of three independent experiments. Unpaired *t* test: *** *p* < 0.001; ** *p* < 0.01; * *p* < 0.05.

Increasing ROS levels within the cells could induce the activation of some antioxidant pathways to revert the oxidative condition. One of the main pathways to counteract oxidative cellular damage is Akt/Nrf2 pathway. An increase in p-Akt/Akt protein ratio is recorded 24 h after two cycles application on 150 µM H_2_O_2_-stimulated cells compared to UT1 (Figure 6A); however, no statistically significant changes are recorded in Nrf2 protein expression levels (Figure 6B). On the contrary, 24 h after T0+T24 CMFs stimulation, a similar statistically significant trend of p-Akt/Akt protein ratio is recorded in H_2_O_2_-stimulated ECs compared to UT2 (Figure 6A) and, at the same time, a significant increase in Nrf2 protein levels is detected (Figure 6B).

To confirm the antioxidant capacity of CMFs application, mainly when T0+T24 is applied, a specific antioxidant assay was performed, measuring the Total Antioxidant Capacity (TAC) within the cells (cell lysate) and released in the supernatants. The application of two consecutive cycles, with respect to UT1, does not affect EC TAC in a statistically significant manner, measured 48 h after the stimulation, either in cell lysates (Figure 6C) or in cell supernatants (Figure 6D). On the contrary, applying T0+T24 condition, a significant increase in TAC is detectable compared to UT2 in both cell lysate and cell supernatants with a similar trend (Figure 6C,D).

## 3. Discussion

CMF application is a new promising non-invasive strategy to treat several pathological conditions, such as diabetic foot, burns, edema resorption, muscular lesions, and many others. Compared to systems emitting Electromagnetic Fields (EMFs) that can act at several frequencies and intensities (low, intermediate, and high), CMFs are more advantageous because the maximum biological efficacy is obtained within 1–250 Hz of frequency range and from 0.1 to 2.5 G of intensity. Thus, CMFs act at extremely low frequency, using minimal energy input to induce a cellular effect, preventing tissue heating and mitigating the risk of intolerance to electrical currents which could induce tissue damage [24]. Nevertheless, the biological impact of these emerging magnetic fields on cellular systems remains largely unexplored. In the last two years, great attention was given to the discovery of CMF cellular effect, evidencing the great impact of this system in the modulation of oxidative stress and inflammation. Indeed, one of the first published papers shed light on how CMFs can alleviate the stress caused by diabetic conditions in fibroblasts and monocytes. This effect was achieved by lowering ROS and some pro-inflammatory cytokines and significantly increasing the levels of anti-inflammatory cytokines [17]. A second paper highlighted the capability of CMFs to modulate several macrophages’ polarization markers, reducing superoxide anion levels and promoting Dental Pulp Stem Cells (DPSCs) differentiation [18]. In addition, our group also brought attention to the effect of CMFs on the endothelial compartment, since the vascular system plays a key role in coordinating the fundamental healing processes necessary for tissue regeneration and oxidative stress fighting. CMF application, in fact, exerts a positive effect in modulating the angiogenic process, acting on several angiogenic steps [20]. Thus, the present research focuses on the effect of CMFs in counteracting an oxidative stress-stimulated ECs environment. With the aim of establishing an oxidative environment, ECs underwent two different stimulating conditions to further select the more suitable one: 150 µM and 300 µM H_2_O_2_ for 3 h. H_2_O_2_ is known to be a key regulator in oxidative metabolism: physiological concentrations (between 1 and 10 nM) act daily in the cells as a signal molecule to support cellular response, inducing the occurrence of an oxidative stress namely eustress. However, in pathological conditions, in which concentrations of H_2_O_2_ exceeding 100 nM are detectable, a different type of oxidative stress occurs, defined as a distress [25]. Our system aims at inducing a distress environment reflecting the conditions experienced by cells when a pathological situation occurs; however, without affecting cell viability in a consistent manner. In this experimental model, flow cytometry analysis confirms increased levels of ROS when ECs are exposed to both concentrations of H_2_O_2_, with a similar trend between them; this result lets us define an efficient oxidative environment. The application of CMFs, in two consecutive cycles or in two cycles spaced 24 h apart, consistently reduce ROS amounts, confirming what was previously found in different cellular systems [17,18] and further demonstrating the crucial point consisting in an appreciable CMF capability to modulate oxidative stress and inflammation. It is also widely known that increased ROS levels influence, in turn, cellular metabolic state, relevant to mitochondrial metabolic activity, either as an indirect measure of viability or in terms of cytotoxicity [26,27]. Indeed, in our system, a relevant reduction in mitochondrial metabolic activity, highlighted by MTT results, along with an increased cytotoxicity, evidenced by LDH levels, are detected when cells are exposed to H_2_O_2_ because of ROS production. However, as previously reported, a distress condition that does not impair cell viability by more than 30%, was selected. CMFs already demonstrated a positive capability to modulate EC mitochondrial metabolic activity [20], as reported in different cellular systems [28,29]: here we corroborate this effect, highlighting that in a stressful condition, the application of this non-invasive tool also appears efficient in cell recovery induction. This data is further confirmed by LDH results: as also demonstrated on DPSCs and LPS-stimulated macrophages [18], CMFs can revert cytotoxicity induced by specific stressful stimuli, supporting the evidence that this system is capable of fighting the stressful environment across different cell models. With the idea of maintaining a distress environment that is able to trigger molecular changes within the cells, the authors then selected only one stressful stimulation (150 µM H_2_O_2_) to perform further experiments, coherent with the literature information [22,23].

When increased ROS levels are detectable, physiological cellular responses are activated. The first protection level is the stimulation of antioxidant molecules synthesis; among them, SODs enzymes are essential to protect cells from ROS-induced oxidative damage [30]. Our results point out that, based on the interval time elapsed between CMF cycle application, two prevalent SODs isoforms, cytosolic SOD-1 and mitochondrial SOD-2, are differently expressed: when two consecutive cycles are applied, within the 24 h the oxidative stress environment induced by CMFs stimulates SOD-2 protein expression, presumably acting on the mitochondrial compartment. On the contrary, when the two cycles are spaced 24 h apart (T0+T24), cytosolic SOD-1 protein is mainly expressed.

Increased ROS levels and distress conditions can act at the mitochondrial level, triggering pro-apoptotic pathways as a possible mechanism to fight oxidative stress, promoting increased expression of p53 and Cytochrome C and culminating in Caspase-3 activation and apoptosis induction [31]. This mechanism can be related to pathological events if the distress condition is persistently activated to the point that cells become unable to restore their normal function, leaving apoptosis as the only possible outcome. On the contrary, in several contexts, this process can be considered physiological, activated by cells to fight stress conditions and to stimulate cellular turnover and renewal [10]. Two consecutive cycles of CMFs seem to positively promote cellular turnover through mitochondrial stimulation: indeed, within 24 h after CMF stimulation, a simultaneous increased expression of mitochondrial SOD-2 and of some pro-apoptotic proteins such as p53 and Cytochrome C occurs. However, there is no activation of apoptotic cascade: Caspase-3 is not cleaved, and 48 h after stimulation the Annexin/PI results demonstrate that there is no apoptosis induction, only a slight increase in the apoptotic cells percentage, compatible with a stimulation of cellular renewal. Considering that the role of SOD-2 in protecting cells from apoptosis and promoting cellular turnover has been identified [11,32], it is plausible that the application of two cycles protects ECs from distress through an increased expression of SOD-2 which, in turn, mitigates the apoptotic event. To corroborate the hypothesis of cellular renewal induction, results demonstrate a positive promotion of the cell cycle, with an increased percentage of cells in the G2 phase 24 h after CMF application and, simultaneously, an increased number of living cells 48 h after the stimulation. Moreover, a promotion of EC migration is demonstrated, further confirming that this application of CMFs supports cellular turnover, renewal, and healing processes.

A second possible mechanism to fight oxidative stress occurrence is represented by the synthesis of antioxidant molecules. Among several oxidant-protective agents, cytosolic SOD-1 and Nrf2 emerged. SOD-1 is an enzyme that mainly regulates the antioxidant response within the cells, not only promoting the conversion of superoxide anion free radical into hydrogen peroxide and molecular oxygen [33] but also acting as a nuclear transcription factor that binds promoter regions to modulate the expression of genes related to oxidative stress response [34]. Nrf2 is a further transcription factor located in the cytoplasm in association with its inhibitor Keap1; when oxidative stress occurs, through the activation of some upstream pathways that converge on Nrf2, such as PI3K/Akt pathway, Nrf2 releases Keap1, which is, in turn, degraded by the proteasomal complex, leading Nrf2 to translocate into the nucleus to bind antioxidant response elements (AREs) to promote cellular antioxidant responses [35,36]. The application of two CMF cycles spaced 24 h apart (T0+T24) seems to revert the distress condition in ECs by regulating these antioxidant molecules: indeed, an increased expression of cytosolic SOD-1 is recorded in addition to an increased expression of Akt/Nrf2. To understand the role and the sequence in which the events occur, more explorations are required; it can be hypothesized that T0+T24, increasing the levels of SOD-1 and Nrf2, can protect cells from the apoptotic events, delaying or suppressing them, and promoting cellular turnover, as already reported in the literature [37]. Indeed, our results demonstrate that the pro-apoptotic proteins are lower in CMF stimulated ECs compared to control, and there is no apoptosis induction, as the flow cytometry analysis reveals, while an increased number of living cells is recorded. On the other side, both SOD-1 and Nrf2, acting as transcription factors, can promote the synthesis and the release of antioxidant elements: our results, in fact, demonstrate that when T0+T24 is applied, 48 h after the stimulation, an increased concentration of antioxidant elements is detected, reported as Total Antioxidant Capacity, in both cell lysates and supernatants, exerting protective effects in autocrine and paracrine ways, respectively.

## 4. Materials and Methods

### 4.1. Cell Culture

The human umbilical vein cell line EA.hy926 was obtained from ATCC (LGC Standards S.r.l., Milan, Italy). They were established by fusing primary human umbilical vein cells with a thioguanine-resistant clone of A549 to obtain an immortalized endothelial cell model. Cells were maintained in DMEM High Glucose containing 10% Fetal Bovine Serum (FBS), 1% penicillin/streptomycin, and 4 mM L-glutamine (all purchased from Euroclone S.p.A., Milan, Italy) and incubated at 37 °C with 5% CO_2_.

### 4.2. Cell Treatment and Stimulation

**Figure 7:**
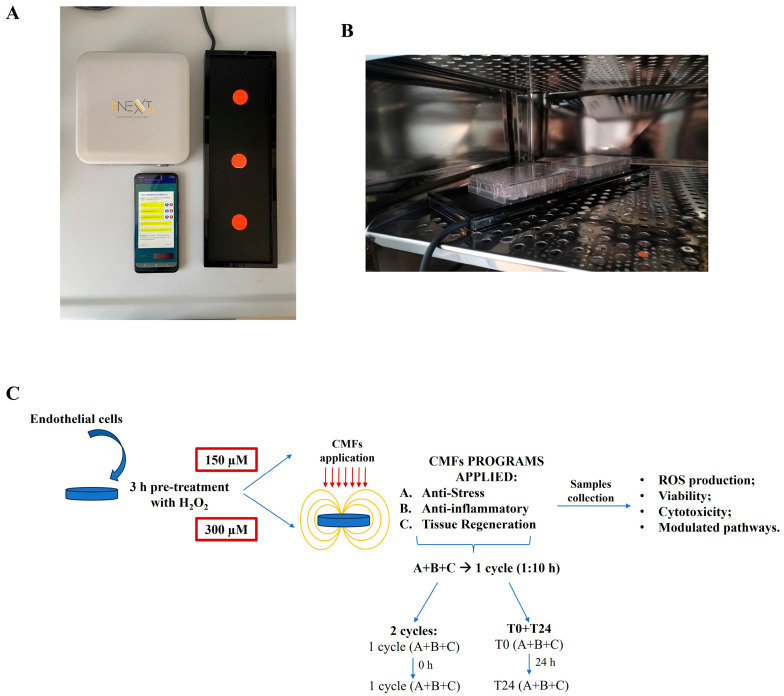
(**A**): Image of CMF emitting device composed of an electronic part, a CMF emitting plaque, where supports with cells are located, and a cellphone. (**B**): Representative image of the emitting plaque located in the incubator with specific cell-seeded support on it. (**C**): Representative image of the study design and of the selected experimental conditions.

Cells, opportunely seeded on the specific required support, were first pre-treated with 150 µM or 300 µM H_2_O_2_ (H_2_O_2_ stock solution 30% *v*/*v*, Sigma-Aldrich, Milan, Italy) for 3 h and then stimulated with CMFs. More precisely, after H_2_O_2_ pre-treatment, culture medium was replaced with a new one and cells were stimulated with the emitting CMF device, Next sx version (M.F.I. Medicina Fisica Integrata, Rome, Italy). The device is composed of an electronic part, a CMF emitting plaque, where supports with cells can be located, and a cellphone to select the specific stimulation program (Figure 7A,B).

Different stimulation programs can be selected through the cellphone (Table 1A), resulting in a tissue specific therapeutic protocol.

When the specific program is launched, the emitting plaque starts to generate CMFs, defined as magnetoelectric fields with a complex geometric configuration of multiple harmonics, whose specific characteristics, listed in Table 1B, depend on the chosen program [24]. The integration and sequential arrangement and alignment of these parameters (frequency, intensity, time, and type of waveform) induce activation of biological systems.

The three programs selected for our purpose were anti-stress program (A), anti-inflammatory program (B), and regenerative tissue program (C), with a treatment duration of approximately 1 h and 10 min, thus indicating that, for each program, the frequency, intensity, interval time, and waveform variation convey precise packets of information, in alignment with the specific cellular system, in order to obtain anti-oxidative stress response for program A, anti-inflammatory response for program B, and stimulation of tissue regeneration for program C.

In our protocol, the combination of the three programs was repeated at 2 different time intervals: in the first condition, two consecutive cycles of A+B+C were applied; in the second condition, two cycles of A+B+C were repeated with an interval of 24 h (T0+T24) (Figure 7C). Control cultures were arranged in the incubator without receiving stimulation: UT1 is the control of 2 cycles, UT2 is the control of T0+T24, being in culture 24 h more than UT1. An additional control, UT, was prepared: this control, with respect to UT1 and UT2, does not receive H_2_O_2_ pre-treatment and it was used to assess the capability of H_2_O_2_ stimulation to establish an oxidative stress environment.

### 4.3. Flow Cytometry Analysis of ROS Production

ROS generation was measured using the probe 2′,7′-dichlorodihydrofluorescein diacetate (H_2_DCFDA) (Molecular Probes, Invitrogen, Life Sciences Division, Milan, Italy). Cells were incubated with probe 1 h at 37 °C: H_2_DCFDA passively diffuses into the cells and after cleavage of the acetate groups by intracellular esterases, if ROS are produced by cells, the oxidation process converted the non-fluorescent H_2_DCFDA into the highly fluorescent 2′,7′-dichlorofluores, detected by an FC500 cytometer with an FL1 detector in a linear mode using the CXP 2.2 software (Beckman Coulter, Brea, CA, USA). The mean fluorescence intensity (MFI) ratio was obtained by histogram statistics and provided to quantify ROS production. Dead cells were excluded from analysis by propidium iodide (PI) staining (5 µg/mL) (Sigma–Aldrich, Milan, Italy). At least 15,000 events for each sample were acquired.

### 4.4. Cell Metabolic Activity Assay (MTT)

An indirect measure of EC viability after exposure to the aforementioned experimental conditions of H_2_O_2_ and CMFs was performed through a metabolic activity test (MTT): viable cells convert MTT into violet formazan salts, then dissolved by DMSO. After 24 and 48 h, the treatment culture medium was removed and replaced by a fresh one containing 10% of MTT (Merck Life Science, Milan, Italy). After 4 h of incubation, formazan salts were dissolved in DMSO for 30 min at 37 °C. Absorbance was spectrophotometrically read by a microplate reader at 540 nm wavelength (Multiskan GO, Thermo Scientific, Waltham, MA, USA). The obtained values of UT1 and UT2 were normalized on UT untreated cell values, while values obtained from the CMF treatment (2 cycles and T0+T24) were normalized on UT1 and UT2, respectively. Results are expressed as a percentage of cell viability.

### 4.5. Lactate Dehydrogenase (LDH) Assay

CytoTox 96 Non-Radioactive Assay (Promega Corporation, Fitchburg, WI, USA) was used to quantify the amount of LDH released in the culture media as direct information of system cytotoxicity. Supernatants of each condition were mixed with LDH reaction mixture (1:1) and incubated for 30 min in the dark. An amount of 50 μL of stop solution was added to block the reaction and the absorbance was read at 490 and 690 nm wavelength by means of Multiscan GO microplate reader (Thermo Scientific, Waltham, MA, USA). Released LDH was normalized on MTT and data are presented as percentage of LDH released.

### 4.6. Western Blot Analysis

EA.hy926 cells were seeded in a 6-well plate at 200,000 cells/well, and 24 h after the specific CMFs stimulation, cells were collected to extract and quantify proteins, as previously described [38]. Lysates were run on polyacrylamide gel by electrophoresis and transferred to nitrocellulose membranes. The membranes were probed overnight with anti-tubulin (dilution 1:5000), anti-Cytochrome C, anti-Nrf2 (all diluted 1:200, all from Santa Cruz Biotechnology, Santa Cruz, Dallas, TX,, USA), anti-p53, anti-Akt, anti-p-Akt, anti-Caspase 3 (all diluted 1:1000 and all from Cell Signaling Technology, Danvers, MA, USA), anti-SOD-1 and anti-SOD-2 (both diluted 1:1000 and both from Invitrogen, Life Sciences Division, Milan, Italy). The day after, membranes were probed in the presence of specific IgG horseradish peroxidase (HRP)-conjugated secondary antibodies (Calbiochem, Darmstadt, Germany) and the ECL detection system (LiteAblot Extend Chemiluminescent Substrate, EuroClone S.p.a., Milan, Italy) was used to reveal immunoreactive bands by using a digital imaging system Alliance 4.7 (UVITEC, Cambridge, United Kingdom). The densitometric values of internal tubulin were used to standardize the obtained data.

### 4.7. Detection of Apoptosis and Necrosis by Flow Cytometry

A total of 48 h after the specific stimulation, ECs were collected together with the supernatants. Cells were stained with Annexin V and Propidium Iodide (PI) (eBioscience, Thermo Fisher Scientific, Waltham, MA, USA) in order to discriminate apoptotic and necrotic cells, according to the manufacturer’s instructions. Samples were probed in binding buffer and of Annexin V FITC (197 µL +3 µL, respectively) in the dark for 15 min at room temperature. After that, the volumes were doubled, and the cells were centrifuged and resuspended in binding buffer with PI. A CytoFlex flow cytometer (Beckman Coulter, Brea, CA, USA) was used to quantify fluorescence. A 530/30 bandpass filter was used to analyze FITC fluorescence (FL-1), and a 650 nm long pass filter for PI fluorescence (FL-3). Data were acquired (2 × 10^4^ events/sample) and analyzed by means of the CytExpert 5.0 Software (Beckman Coulter, Brea, CA, USA). Viable cells percentage (Annexin V-; PI-) were revealed in the lower left quadrant (unstained) of density plots, cells in apoptosis in lower right quadrant (Annexin V+/PI-), cells in late apoptosis in upper right quadrant (Annexin V+/PI+), and cells in necrosis in upper left quadrant (Annexin V-/PI+).

### 4.8. Trypan Blue Dye Exclusion Test

A total of 24 and 48 h after the specific stimulations, cells were washed with PBS, trypsinized and processed for Trypan blue dye exclusion test, which selectively discriminates the live cells from the blue colored dead cells, counted in a Burker chamber. The number of live cells is reported as number of cells/mL of solution.

### 4.9. Cell Cycle Analysis

ECs were stimulated for 3 h with H_2_O_2_ 150 µM followed by 2 cycles and T0+T24 treatment. A total of 24 h after CMFs treatment, cells were collected and fixed overnight with cold ethanol 70% *v*/*v*. The day after, samples were stained overnight at 4 °C in the dark by using 300 µL of a solution containing PBS, Rnase 100 µg/mL (stock solution 10 mg/mL in 10 mM sodium acetate buffer, pH 7.40), and propidium iodide (PI) 10 µg/mL (stock solution 1 mg/mL in water) (Sigma Aldrich, MI, USA). PI fluorescence was detected by a flow cytometer equipped with a 488 nm laser (CytoFlex flow cytometer, Beckman Coulter, Brea, CA, USA) on the FL-3 channel. CytExpert Software (Beckman Coulter, Brea, CA, USA) was used to analyze the collected 15,000 events/sample. The distribution of cells in the G1, S, or G2 phase of the cell cycle were calculated using the ModFit LT™ 5.0 Software (Verity Software House, Topsham, ME, USA) and expressed as percentage of cells.

### 4.10. Wound Healing

EA.hy926 cells were seeded in 6-well plates at 250,000 cells/well and once cell monolayer reached 70–80% of confluence, they were stimulated for 3 h with 150 µM H_2_O_2_ and then scratched by using a p200 pipet tip (Falcon, Corning Incorporated, New York, NY, USA). Debris formation followed by cell monolayer were removed by washing wells with PBS and culture media and then cells were stimulated with the specific CMF treatment. Images were acquired after 0, 16, 24, and 48 h of stimulation with an inverted light microscope Leica DMI1 (Leica Cambridge Ltd., Cambridge, UK) with a Leica MC120 HD camera (Leica Cambridge Ltd., Cambridge, UK) to obtain computerized images. The migratory capability of cells was evaluated considering T0 (0 h after stimulation) as the starting point and the percentage of wound closure was normalized on the appropriate T0 of each condition.

### 4.11. Total Antioxidant Capacity (TAC) Assay

ECs were seeded in a 6-well plate at a cell density of 200,000 cells/well. After the specific stimulations with H_2_O_2_ and CMFs, cell pellet and cell supernatants were both collected for each condition to measure TAC in cell lysate and TAC released, respectively. Briefly, 20 µL of cell lysate or cell supernatants were plated in a well of 96-well plate and 100 µL of a specific Reaction Mix was added and maintained for 10 min at room temperature, following manufacturers’ instructions. Absorbance was read at 570 nm wavelength by using a spectrophotometer (Multiskan GO, Thermo Scientific, Waltham, MA, USA) and values were interpolated with a standard curve to obtain the concentration of antioxidant molecules in the samples (µM) and normalized on MTT values.

### 4.12. Statistical Analysis

GraphPad software 10.3.0 was used to statistically analyze the data sets. Analysis was carried out using ANOVA and Student’s *t* test: two conditions were compared using unpaired *t test*; multiple groups were analyzed using one-way ANOVA followed by Tukey’s or Dunnett’s post hoc analyses. The results are presented as the mean values ± SD. Values of *p* ≤ 0.05 were considered statistically significant.

## 5. Conclusions

Vascular system is of fundamental importance to influence the healing and regeneration of the surrounding tissues, but also to prevent several cardiovascular diseases. CMF application is confirmed to be a promising non-invasive tool to treat several pathological conditions, presumably also thanks to the effect on the endothelial compartment. The *in vitro* model proposed in this research, represented by ECs, which underwent oxidative distress followed by CMF treatment, demonstrates and confirms the favorable medical application of the system. In particular, when two consecutive cycles are applied, the oxidative stress is reverted through an increased expression of SOD-2 that probably controls cellular renewal, reining an altered apoptosis induction and promoting cellular migration and turnover. These results let us hypothesize that this CMFs application could be suitable in pathological conditions in which quick and efficient tissue regeneration is required, such as wound conditions (diabetic foot, post-surgery recovery, burn wounds, and so on). When T0+T24 is applied, oxidative stress is maintained under control mainly through the expression of SOD-1 and Nrf2, culminating in an increased TAC response, making this CMFs application suitable in pathological situations in which an antioxidant and anti-inflammatory effect is required (for instance, it could be apply in edema resorption, muscular or tendon lesions). Taking into account all the limitations of the study, these preliminary data can be considered, promising to move on with further and more in-depth investigations.

## Figures and Tables

**Table 1:** **A**. List of available programs that can be selected on the cellphone. **B**. General characteristics of CMF programs.

**A**	
**Type of Programs**	
**1.** Edema	
**2.** Anti-inflammatory	
**3.** Muscles contractures	
**4.** Anti-stress	
**5.** Chronic pain treatment	
**6.** Analgesic (localized pain)	
**7.** Anti-bacterial	
**8.** Muscles regeneration	
**9.** Tendon regeneration	
**10.** Bone regeneration	
**11.** Tissue regeneration	
**12.** Nervous system regeneration	
**13.** Jet Lag	
**B**	
**Program-Related CMF Parameters**	**Characteristics**
Frequencies	1–250 Hz
Intensities	1–250 µT
Interval Times	1–4 min each step (not exceeding 30 min/program)
Type of waveform with harmonic enrichment	square, sinusoidal, impulsive, triangular, trapezoidal

## Data Availability

Data are contained within the article.
