# Correction: Ricci et al. SOD-1/2 Involvement in the Antioxidant Molecular Events Occurring upon Complex Magnetic Fields Application in an *In Vitro* H_2_O_2_ Oxidative Stress-Induced Endothelial Cell Model. *Int. J. Mol. Sci.* 2025, *26*, 8600

**DOI:** 10.3390/ijms27052387

**Published:** 2026-03-04

**Authors:** Alessia Ricci, Susi Zara, Viviana di Giacomo, Marialucia Gallorini, Monica Rapino, Natalia Di Pietro, Alessandro Cipollina, Adriano Piattelli, Amelia Cataldi

**Affiliations:** 1 Department of Pharmacy, “G. d’Annunzio” University of Chieti-Pescara, 66100 Chieti, Italy; alessia.ricci@unich.it (A.R.); susi.zara@unich.it (S.Z.); viviana.digiacomo@unich.it (V.d.G.); marialucia.gallorini@unich.it (M.G.); 2 Unit of Chieti, Genetic Molecular Institute of CNR, “G. d’Annunzio” University of Chieti-Pescara, 66100 Chieti, Italy; monica.rapino@unich.it; 3 Department of Medical, Oral and Biotechnological Sciences, “G. d’Annunzio” University of Chieti-Pescara, 66100 Chieti, Italy; natalia.dipietro@unich.it; 4 Independent Researcher, 92019 Sciacca, Italy; alexandros1960@libero.it; 5 School of Dentistry, UniCamillus-Saint Camillus International University of Health and Medical Sciences, 00131 Rome, Italy; apiattelli51@gmail.com; 6 Ud’A Techlab, “G. d’Annunzio” University of Chieti-Pescara, 66100 Chieti, Italy

The following reference [24] has been retracted and has to be removed from the original publication [1]:

24. Jia, F.; Mou, L.; Ge, H. Protective effects of ginsenoside Rb1 on H_2_O_2_-induced oxidative injury in human endothelial cell line (EA.hy926) via miR-210. *Int. J. Immunopathol. Pharmacol.* **2019**, *35*, 20587384211040399.

The scientific content of [24] could not be replaced with any other publication. Therefore, the authors decided to remove reference [24]. With this correction, the order of some references has been adjusted accordingly.

The authors state that the scientific conclusions are unaffected. This correction was approved by the Academic Editor. The original publication has also been updated.
